# Development of a triple NanoPCR method for feline calicivirus, feline panleukopenia syndrome virus, and feline herpesvirus type I virus

**DOI:** 10.1186/s12917-022-03460-9

**Published:** 2022-10-27

**Authors:** Jingfei Ye, Zhijie Li, Fei Yan Sun, Li Guo, Erkai Feng, Xue Bai, Yuening Cheng

**Affiliations:** 1 Institute of Special Animal and Plant Sciences, Chinese Academy of Agricultural Sciences, Changchun, 130112 China; 2 Jilin Agricultural Science and Technology University, Jilin, 132109 China

**Keywords:** NanoPCR, Detection, FCV, FPV, FHV-1

## Abstract

**Background:**

Feline calicivirus (FCV), Feline panleukopenia virus (FPV), and Feline herpesvirus type I (FHV-1) are the three most common pathogens in cats, and also are the main pathogens leading to the death of kittens. Here, by a combination of gold nanoparticles and conventional PCR, we established a novel triple NanoPCR molecular detection method for clinical detection.

**Results:**

The triple NanoPCR molecular detection is able to detect 2.97 × 10^1^copies/μL FCV recombinant copies plasmid per reaction, 2.64 × 10^4^copies/μL FPV recombinant copies plasmid per reaction, and 2.85copies/μL FHV-1 recombinant copies plasmid per reaction at the same time. The sensitivity of each plasmid is 100 times, 10 times, and 100 times higher than conventional PCR, respectively. The clinical results showed that among the 38 samples, the positive rates of FCV, FPV, and FHV-1 in a NanoPCR test were 63.16, 31.58, and 60.53%, while in a conventional PCR were 39.47, 18.42, and 34.21%.

**Conclusions:**

In this report, it is the first time that NanoPCR assays are applied in the detection of FCV, FPV, and FHV-1 as well. This sensitive and specific NanoPCR assay can be widely used in clinical diagnosis and field monitoring of FCV, FPV, and FHV-1 infections.

## Background

Feline calicivirus (FCV) is the most common pathogen that can cause feline respiratory disease which often infects kittens under the age of one, and is highly prevalent in clinical practice. After infected with FCV, most kittens develop acute respiratory disease and oral ulcers, while mature cats develop mild or no symptoms [1]. Due to the high variability and high contagiousness of FCV, feline calicivirus disease has gradually become one of the most important diseases threatening the health of felines.

Feline panleukopenia syndrome virus, also known as feline parvovirus (FPV). All felines are susceptible to FPV, especially kittens under the age of one. Feline parvovirus is widespread on the account of its high infectivity and strong resistance to environmental factors [2]. The incubation period is 3 to 4 days after infection, and the infected animals would have fever, depression, and anorexia. Most infected cats vomit, usually with yellow vomit sounds strange, and are extremely dehydrated [3].

Feline herpesvirus type I (FHV-1) has a very high species specificity, pet cats are the main susceptible hosts, especially for those 4–6 weeks old kittens. Acute infection in these kittens can cause severe clinical symptoms and its mortality is as high as 50%. Mature cats generally show no obvious clinical symptoms after BEING infected with FHV-1, but the virus would replicate in the upper respiratory tract, oral mucosa, conjunctiva, cornea, turbinate and other parts, and continuously detoxifies [4].

Nanoparticle-assisted PCR (NanoPCR) is a novel method for rapidly amplifying DNA. It is to amplify nucleic acid through adding gold nanoparticles to conventional PCR reactions [5]. Since gold nanoparticles have a higher affinity for single-stranded DNA than for double-stranded DNA, they can improve the significant specificity of PCR reactions and reduce mismatches [6]. The excellent heat transfer properties enable NanoPCR to have a greater heating/cooling rate and sensitivity compared with conventional PCR reactions, thereby shortening the reaction time and improving the amplification efficiency [7].

Clinically, since the above three viruses often co-infect, and felines are generally susceptible to them, a rapid and sensitive detection method should be established to detect as soon as possible and control the spread of the disease in time.

On the purpose to have a convenient, fast, and effective detection of these three pathogens, our laboratory uses a combination of gold nanoparticles with conventional PCR molecular detection. On the basis of triple PCR and by the character of gold nanoparticles, the detection method with more effectiveness and higher sensitiveness is built and guarantees the early diagnosis of feline diseases.

## Result

### Optimization of NanoPCR conditions

**Fig. 1:**
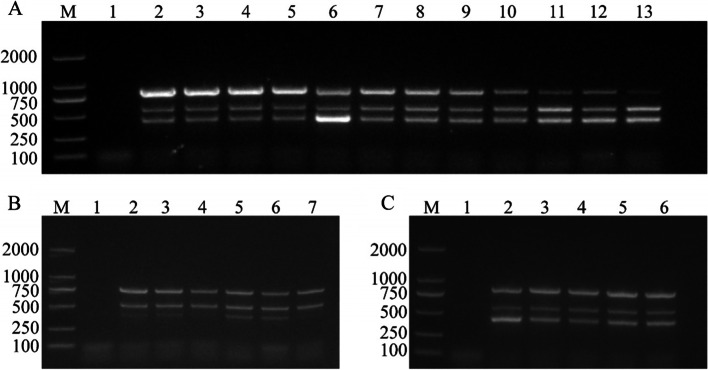
Optimization of annealing temperature (**A**), gold nanoparticle concentration (**B**), and gold nanoparticle diameter (**C**). Lane M: Trans2K DNA Marker (TransGen Biotech, BM101). **A** Lane 1: Negative control, lane 2–13: Annealing temperatures of 49°C, 49.4°C, 50.2°C, 51.4°C, 53°C, 54.2°C, 55.6°C, 56.2°C, 58.4°C, 59.5°C, 60.4°C, 61°C. **B** Lane 1: Negative control, lane 2–7: The gold nanoparticles in volumes from 0.5 μL to 3 μL in increments of 0.5 μL. **C** Lane 1: Negative control, lane 2–6: The gold nanoparticles with diameters of 20 nm, 30 nm, 40 nm, 60 nm and 100 nm

Based on conventional PCR, we optimized the optimum concentration, diameter, and annealing temperature of the added gold nanoparticles. The results show that the brightness of the three templates can be well balanced at 53°C (Fig. 1A). Using this annealing temperature, the band density was found to be higher with the addition of 2 μL (Fig. 1B) of 60 nm (Fig. 1C) gold nanoparticles.

According to the optimized results, the optimal reaction system was determined to be 25 μL, containing 12.5 μL of 2 × Rapid Taq Master Mix (Vazyme Biotech, P222), 0.5 μL of forward and reverse primers (10 μM), 2 μL of 60 nm gold nanoparticles, 1 μL of extracted nucleic acid or standard plasmid, ddH_2_O to make up 25 μL. Cycle times and temperatures were the same as for conventional PCR reactions.

### Sensitivity of NanoPCR

**Fig. 2:**
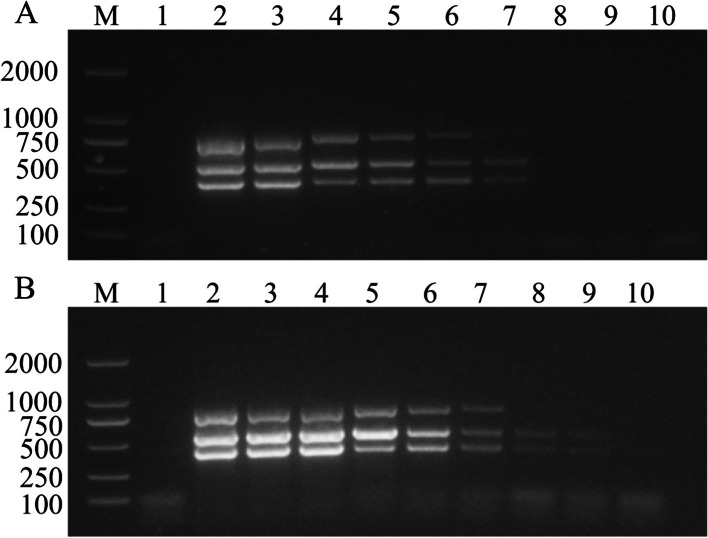
The sensitivity of conventional PCR (**A**) and NanoPCR (**B**) to detect FCV, FPV, FHV-1 plasmid DNA. Lane M: Trans2K DNA Marker (TransGen Biotech, BM101). Lane 1: Negative control. Lanes 2–10: FCV plasmid DNA is 2.97 × 10^8^copies/μL to 2.97copies/μL, 10-fold dilution; FPV plasmid DNA is 2.64 × 10^9^copies/μL to 2.64 × 10^1^copies/μL, 10-fold dilution; FHV-1 plasmid DNA is 2.85 × 10^7^copies/μL to 2.85 × 10^−1^copies/μL, 10-fold dilution

The results of triple NanoPCR showed that the detection limits of FCV, FPV, and FHV-1 were 2.97 × 10^1^copies/μL, 2.64 × 10^4^copies/μL, and 2.85copies/μL, and was 100 times, 10 times and 100 times sensitive than that of conventional PCR (Fig. 2).

### Specificity analysis of NanoPCR

**Fig. 3:**
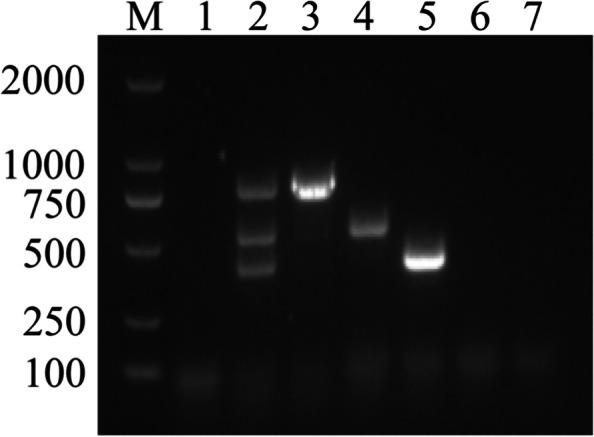
NanoPCR specificity analysis. Lane M: Trans2K DNA Marker (TransGen Biotech, BM101). Lane 1: Negative control. Lanes 2–5: FCV, FHV, FHV-1 nucleic acid mix. Lane 2: FCV, FPV, FHV primers. Lane 3: FPV primers. Lane 4: FHV-1 primer. Lane 5: FCV primers. Lane 6: FIPV nucleic acid. Lane 7: RABV nucleic acid

Use primers of FCV, FPV and FVH-1 to amplify the mixed sample of FCV, FPV and FHV-1. The results are shown in lanes 3–5 of Fig. 3. NanoPCR reactions were performed on nucleic acids of Feline infectious peritonitis virus (FIPV) and Rabies virus (RABV). The final results showed that the specificity of each primer was good, and there was no cross-amplification reaction (Fig. 3).

### Clinical sample testing

**Fig. 4:**
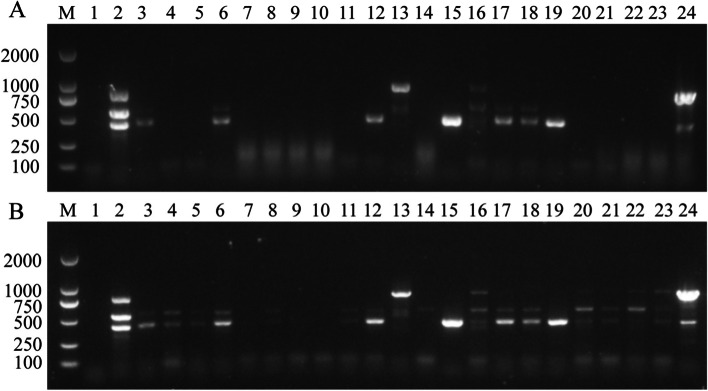
Comparison of conventional PCR (**A**) and NanoPCR (**B**) to detect FCV, FPV, and FHV-1 in clinical samples. Lane M: Trans2K DNA Marker (TransGen Biotech, BM101). Lane 1: Negative control. Lane 2: FCV, FPV, FHV-1 plasmid DNA as template. Lanes 3–24: Cat nasal swabs and fecal samples from the clinic

Thirty-eight samples of cat fecal and cat nasal swabs were collected from pet hospitals. All 38 samples were detected using conventional PCR and NanoPCR. The results showed that 15 FCV positive samples, 7 FPV positive samples and 13 FHV-1 positive samples were detected by conventional PCR, while 24 FCV positive samples (positive rate was 63.16%), 12 FPV positive samples (positive rate was 31.58%) and 23 FHV-1 positive samples (positive rate was 60.53%) were detected by NanoPCR. Eight positive samples were randomly purified and sequenced. Sequencing results showed that the amplified product of NanoPCR was very similar to the sequence of the reference strain. Part of the results are shown in Fig. 4, statistical results are shown in Tables 1, 2 and 3.

**Table 1:** Sensitivity comparison of NanoPCR and conventional PCR assays for the detection of FCV in fecal samples and nasal swab samples

NanoPCR	Conventional PCR		
	Positive	Negative	Total
Positive	15	9	24
Negative	0	14	14
Total	15	23	38

The conventional PCR positive rate of FCV: 15/38 = 39.47%; The Nano PCR positive rate of FCV: 24/38 = 63.16%

**Table 2:** Sensitivity comparison of NanoPCR and conventional PCR assays for the detection of FPV in fecal samples and nasal swab samples

NanoPCR	Conventional PCR		
	Positive	Negative	Total
Positive	7	5	12
Negative	0	26	26
Total	7	31	38

The conventional PCR positive rate of FPV: 7/38 = 18.42%; The Nano PCR positive rate of FPV: 12/38 = 31.58%

**Table 3:** Sensitivity comparison of NanoPCR and conventional PCR assays for the detection of FHV-1 in fecal samples and nasal swab samples

NanoPCR	Conventional PCR		
	Positive	Negative	Total
Positive	13	10	23
Negative	0	15	15
Total	13	25	38

The conventional PCR positive rate of FHV-1: 13/38 = 34.21%; The Nano PCR positive rate of FHV-1: 23/38 = 60.53%

## Discussion

As the most common pathogens among felines, FCV, FPV, and FHV-1 are widespread in unvaccinated cats. Although most mature cats have only mild clinical symptoms or are asymptomatic, their continuous carrying and excretion of virus threaten the life of newborn cats [8]. It is significant to have the efficient, sensitive, and convenient detection measurements to prevent disease outbreaks. In this study, a triple PCR for three pathogens, FCV, FPV, and FHV-1 was established, and combined with gold nanoparticles, a more sensitive triple NanoPCR was established. The gold nanoparticles are self-made in our laboratory and are used in the rapid detection of diseases after being tested by a nanoparticle size analyzer and quality identification of nano-gold.

The triple PCR method does not simply stack the three primers for amplification, it considered the annealing temperature of the three primers and whether the corresponding fragment sizes are appropriate at the beginning of the design. When establishing a multiplex PCR, it is also necessary to adjust the concentration of the positive template in time. In the experiments, it is found that when the concentrations of the three positive templates were in the same order of magnitude, the addition of nanoparticles would significantly increase the band brightness of FCV and FHV-1 during imaging, thereby disturbing the imaging band brightness of FPV. However, when the template concentrations of the three were low, the amplified bands of FCV and FHV-1 still had good brightness. In order to balance this phenomenon, we used different concentrations of templates for the sensitivity test and finally selected 2.64 × 10^10^copies/μL of FPV-positive plasmid, 2.85 × 10^8^copies/μL of FHV-1-positive plasmid, and 2.97 × 10^9^copies/μL of FCV-positive plasmid, mixed as the initial template for triple PCR.

In 2019, Liu et al. [9] established a NanoPCR method for the detection of Bovine respiratory syncytial virus (BRSV) and proved that adding 0.7 μL of gold nanoparticles to conventional PCR could increase the sensitivity of detecting BRSV by 10 times. Applying it to the detection of clinical samples, the results showed that NanoPCR is more sensitive than conventional PCR, and the detection efficiency is more obvious.

Clinically, it is the test strips that are mainly used in detecting FCV, FPV and FHV-1. The test strip method is convenient and quick while it can only work better when there is an amount of virus particles in the samples. The PCR detection can detect the pathogen as soon as possible through exponential nucleic acid amplification when there are a small number of virus particles in the body. RT-qPCR detection is more accurate, but requires professional and delicate operations, complex equipment, and expensive detection prices, and is not suitable for multiple pathogen detection in a single sample.

Since gold nanoparticles are relatively cheap to prepare and have a good thermal conductivity which enables the PCR reaction to reach the target temperature in a shorter time, and the high-efficiency single-strand binding property improves the nucleic acid amplification efficiency. Therefore, efficient heat transfer generates a large number of amplicons and also increases the sensitivity of the reaction.

In summary, the established novel triple NanoPCR is effective for detecting FCV, FPV, and FHV-1. Its sensitivity is higher than conventional PCR, and it can detect at least 2.97 × 10^1^copies/μL of FCV and 2.64 × 10^4^copies /μL of FPV, and 2.85copies/μL of FHV-1. The assay method is specific to FCV, FPV, and FHV-1, and can be applied to the detection of three types of virus molecules. It will play an active role in the detection, prevention, and biosafety to FCV, FPV, and FHV-1 so as to achieve a rapid diagnosis of FCV, FPV, and FHV-1. At the same time, it is also of great significance for the early detection and disease control of subclinical infection. Although the clinical sample of this study is limited, more samples will be evaluated in the future.

## Conclusion

This study established a rapid and highly sensitive triple NanoPCR method for FCV, FPV, and FHV-1. The method combines gold nanoparticles based on the established triplex PCR and simultaneously detects the nucleic acids of the three viruses, and at the same time greatly improves the detection sensitivity of these viruses. The detection limits of NanoPCR for FCV, FPV, and FHV-1 were 2.97 × 10^1^copies/μL, 2.64 × 10^4^copies/μL, and 2.85copies/μL, which were 100 times, 10 times, and 100 times sensitive than conventional PCR, respectively. The detection efficiency of clinical samples is significantly higher than that of conventional PCR. The assay developed in this study can be applied in the field of molecular detection of common feline viral diseases. It has positive significance for the rapid diagnosis of FCV, FPV, and FHV-I in clinical practice can be used for early disease detection, monitoring, and clinical epidemiological investigation, and provides technical support for the comprehensive prevention and control of the three viruses.

## Methods

### Viruses and clinical samples

The purified FCV, FPV, FHV-1, FIPV, and RABV are preserved in our laboratory. Fecal samples and nasal swab samples were obtained from several pet hospitals in Changchun, Jilin province, China. All samples in our laboratory are previously preserved at − 20°C. The samples were taken from the feces and nasal swabs of the experimental animals, and there was no harm to the animals throughout the experiment.

### Viral DNA/RNA extraction

Following the manufacturer’s instructions, viral nucleic acid was extracted from 200 μL of pure viral culture using a viral DNA/RNA extraction kit (Takara, 9766). RNA of FCV, FIPV and RABV were reverse transcribed using a reverse transcription kit (TransGen Biotech, AT311).

### Primer and recombinant plasmid construction

FCV (GenBank: MW804434.1), FPV (GenBank: MZ836373.1) and FHV-1 (GenBank: MT813047) were identified for the reference strain, after aligning the genomes of FCV, FPV, and FHV-1 isolates in publicly available sequence data. Oligo7.0 Primer Analysis software (Molecular Biology Insights, Inc. USA) was used to design primers for the conserved region fragments, and the predicted fragment lengths are 389 bp, 768 bp, and 513 bp. The designed primers are shown in Table 4. The obtained fragment was ligated into pMD-19 T simple (Takara, 3271) as a standard recombinant plasmid and expanded using Trans1-T1 competent cells (TransGen Biotech, CD501). Recombinant plasmids were purified using a plasmid extraction kit (Omega Biotek, D6943), and finally sent to a biological company for sequencing.

**Table 4:** Primers for Amplification of FCV, FPV, FHV-1 by conventional PCR and NanoRCR

Primer name	Sequence (5′ - 3′)	Product (bp)
FCV-F	CAACCTGCGCTAACGTGCTTA	389
FCV-R	TGCAGTAATGGATCCATCATCCG	
FPV-F	CTTTGCCTCAATCTGAAGGAG	768
FPV-R	GAATTGGATTCCAAGTATGAG	
FHV-1-F	AGATTTGCCGCACCATACCTTC	513
FHV-1-R	CCGGGCTTTGAAAACACTGAAT	

### Conventional PCR reaction

Conventional PCR analysis was performed for FCV, FPV, and FHV-1 using primer sets. The total PCR reaction volume was 25 μL, which contained 12.5 μL of 2 × Rapid Taq Master Mix (Vazyme Biotech, P222), 0.5 μL of forward and reverse primers (10 μM), 1 μL of extracted nucleic acid or standard plasmid, and add ddH_2_O up to 25 μL. Following the manufacturer’s instructions, cycle times and temperatures were as follows: 95°C preheat for 3 mins, 95°C for 15 s, 55°C for 15 s, 72°C extensions for 15 s, cycles for 35 times, and the final extension was performed at 72°C for 5 mins, and the PCR product was electrophoresed on a 1% agarose gel.

### Optimization of NanoPCR

Based on conventional PCR, after adding gold nanoparticles, we optimized the annealing temperature of the PCR reaction system, the concentration and size of gold nanoparticles. The annealing temperature ranged from 49°C to 61°C, and the diameters of the gold nanoparticles were 20 nm, 30 nm, 40 nm, 60 nm, and 100 nm. The volume of gold nanoparticles ranges from 0.5 to 3 μL in 0.5 μL increments. All electrophoretic bands were analyzed using ImageJ 1.46r software (National Institutes of Health, Bethesda, MA, USA).

### Sensitivity of NanoPCR

Construct and identify the correct FCV, FPV, and FHV-1 recombination-positive plasmids were diluted with ddH_2_O to 100 ng/μL, and then perform a 10-fold serial dilution. At this time, the range of FCV recombination-positive plasmids was 2.97 × 10^10^copies/μL to 2.97copies/μL, the range of FPV recombination-positive plasmids was 2.64 × 10^10^copies/μL to 2.64copies/μL, and the range of FHV-1 recombination-positive plasmids was 2.85 × 10^10^copies/μL to 2.85copies/μL. We used ddH_2_O as a negative control and electrophoresed the PCR products on a 1% agarose gel.

### Specificity analysis of NanoPCR

The cDNA of FCV, the DNA of FPV, and the DNA of FHV-1 were mixed and amplified using only a single primer set in the established NanoPCR to determine the specificity of the NanoPCR assay. NanoPCR reactions were performed on nucleic acids of Feline infectious peritonitis virus (FIPV) and Rabies virus (RABV) using optimized reaction parameters to test whether NanoPCR had cross-reaction to other viral nucleic acids.

### Clinical sample testing

Thirty-eight samples of cat fecal and cat nasal swabs were collected from pet hospitals in Changchun and stored frozen in our laboratory. The 38 samples were tested using conventional PCR and NanoPCR parallelly. Some samples were taken and sent to a biological company for sequencing and identification.

## Data Availability

The recombinant plasmid sequence we constructed has been uploaded to GenBank. Accession numbers: FCV strain JL18: ON529575; FPV strain CC17: ON529574; FHV-1 strain HB-19: ON529576.

## Abbreviations

FCV: Feline calicivirus
FPV: Feline parvovirus
FHV-1: Feline herpesvirus type I
BRSV: Bovine respiratory syncytial virus
PCR: Polymerase chain reaction
NanoPCR: Nanoparticle-assisted PCR
FIPV: Feline Infectious Peritonitis virus
RABV: Rabies virus
